# Stricture of the Urethra and Enlargement of the Prostate

**Published:** 1901-12

**Authors:** 


**Stricture of the Urethra and Enlargement of the Prostate.** By P. J. Freyer, M.D. Pp. 115. London: Baillière, Tindall and Cox. 1901.

This is a reproduction of clinical lectures delivered in 1900, in an agreeable form, but without an index. The teachings are generally sound, as one would expect from Mr. Freyer, but there are a few points to which we take exception. For instance, we cannot agree that "the friction caused by the urinary stream keeps up irritation, and may cause excoriation and ulceration at the seat of stricture." In the treatment of stricture by dilatation the author advises that if a false passage be present, the surgeon should persist in endeavours to find the true one: we have to assume that this applies to old false passages rather than recent ones.

It surprises us much to find that in using the Tevan-Maisonneuve urethrotome the incision is made and the scar results in the *roof* of the urethra, which is so much the more vascular. Perhaps the author has not fully considered this matter, for we can imagine such usage of the instrument placing the average operator in a dilemma. We have good reasons for knowing that Holt's dilator has not disappeared from practice, as is stated by Mr. Freyer. The suggested treatment of cystitis by irrigation with a *one* per cent. solution of boric acid is probably a verbal slip for a higher figure. The question of total enucleation of the prostate, which has been recently much under discussion, is solved in the writer's report on his case, which says: "After removal of these tumours no trace of the prostate could be felt between a finger in the bladder and one in the rectum, *the portion of the gland not involved in the tumours having been atrophied by pressure.*" Repeating the same report later on (*Brit. M. J.*, 1901, ii. 126), he says: "I could feel that there was no prostatic substance left behind." The title of his paper, "Total Extirpation of the Prostate," was therefore unfortunate, and his claim to a new operation must fail in favour of McGill in this country.

